# Dr. Richards

**Published:** 1833

**Authors:** 


William Richards, M. D. of *Georgetown, Ky.* This gentleman graduated in Lexington, of whose vicinity he was a native, in the spring of 1823. For some years past he had withdrawn from the practice, and cultivated a plantation in the south, residing in Georgetown in the summer. When "down the river" last autumn, he saw much of the Epidemic, and on its appearance in Georgetown, the people sought to avail themselves of the advantages of his experience. Charging nothing for his services, the poor flocked to him for advice, and thus oppressed with business, he sunk under the Epidemic, in the midst of its reign. Dr. R. was a talented physician and an exemplary gentleman.

